# Triage systems for pre-hospital emergency medical services - a systematic review

**DOI:** 10.1186/1757-7241-21-28

**Published:** 2013-04-15

**Authors:** Ingeborg Beate Lidal, Hilde H Holte, Gunn Elisabeth Vist

**Affiliations:** 1 The Norwegian Knowledge Centre for the Health Services, St. Olavs plass, Po Box 7004, OSLO, 0130, Norway; 2 The Norwegian Knowledge Centre for the Health Services, St. Olavs plass, Po Box 7004, OSLO, 0130, Norway

**Keywords:** Triage, Triage system, Pre-hospital, Emergency medical services, Controlled trials, Emergencies, Effect

## Abstract

The emergency medical services (EMS) cover initiatives and services established to provide essential medical assistance in situations of acute illness. Triage-methods for systematic prioritizing of patients according to how urgent patients need care, including triage of requests of acute medical treatment, are adopted in hospitals as well as in the pre-hospital settings. This systematic review searched to identify available research on the effects of validated triage systems for use in the pre-hospital EMS on health outcomes, patient safety, patient satisfaction, user-friendliness, resource use, goal achievement, and the quality on the information exchange between the different settings of the EMS (for example the quality of documentation). The specific research questions were: 1) are pre-hospital triage systems effective, 2) is one triage system more effective than others, and 3) is it effective to use the same triage system in two or more settings of the EMS-chain? We conducted a systematic literature search in nine databases up to June 2012. We searched for systematic reviews (SRs), randomized controlled trials (RCTs), non-randomized controlled trials (non-RCTs), controlled before and after studies (CBAs) and interrupted time series analyses (ITSs). Two persons independently reviewed titles and abstracts, and the same persons read all possibly relevant full text articles and rated the methodological quality where relevant. The literature search identified 11011 unique references. A total of 120 publications were read in full text. None of the identified articles fulfilled our inclusion criteria, thus our question on the effects of pre-hospital triage systems, if one system is better than other systems, and the question on effects of using the same triage system in two or more settings of the EMS, remain unanswered. We conclude that there is an evidence gap regarding the effects of pre-hospital triage systems and the effects of using the same triage system in two or more settings of the EMS. The finding does not mean that pre-hospital triage systems are ineffective, but that we lack knowledge about potential effects. When introducing a new assessment tool in the EMS, it is timely to conduct well-planned studies aimed to assess the effect.

## Introduction

Triage systems are currently used in the emergency medical services (EMS) both in the pre-hospital setting and in hospitals in Scandinavia. Triage systems are methods for systematic prioritizing of patients’ treatment according to how urgent they need care. The triage result should influence the order and priority of emergency treatment, the order and priority of emergency transport, or the transport destination for the patient. In an acute case, triage assessment is usually done in at least one setting of the EMS, and sometimes triage takes place in all relevant settings of the acute chain, i.e. by the emergency telephone responder, by the first ambulance crew on scene, by primary care physician(s), at the emergency clinic, and at the emergency room/emergency department (ED) in a hospital.

Modern approaches to triage assessment of acutely ill or injured patients are usually based on trace and trigger tools for vital signs, and include a systematic questionnaire for each chief complaint and generally physiological findings. The most common triage systems are those for use in the ED developed during the 1990s and 2000s [1,2]. Of these, the Australian Triage Scale (ATS), the Manchester Triage Scale (MTS), the Canadian Emergency Department Triage and Acuity Scale (CTAS), and the Emergency Severity Index (ESI) have disseminated around the world. Systematic triage assessment of all patients according to validated methods is less common in the pre-hospital setting. However, telephone triage utilizes protocols to help sort symptoms presented by the caller and to activate appropriate dispositions [3-6]. Recently, the medical emergency triage and treatment system (METTS) [7], developed in Sweden, introduced METTS-pre specifically for the use in ambulances services [8].

Many countries in Europe, including the Scandinavian countries do not have a national mandatory triage scale. Within these countries, or even within counties, triage systems implemented are considered with respect to how they may fit the local context, for example population, size of hospital, resources, topography etc. In 2002, Göransson et al. conducted a Swedish national survey and revealed that a total of 37 different versions of triage systems were used, and about half of the participating EDs did not use a triage system at all [9]. By 2010, 97% of Swedish EDs had introduced triage scales, and METTS is the triage scale most commonly implemented across the country [10]. Triage systems are used in about 75% (n = 15) of the Danish EDs [4]. In Denmark, the Adaptive process triage (ADAPT) [11] has been reported to be the most frequently used validated triage system, used by 25% of the EDs, while 40% of the EDs used non-validated systems [4]. In Norway, both university hospitals and local hospitals have implemented triage systems or are about to do so, and METTS, MTS and also non-validated triage system seems to be the most common choices.

In 2010, the Swedish Council on Health Technology Assessment (Statens beredning för medicinsk utvärdering; SBU) published a systematic review of the literature on the effect of interventions aimed to improve patient flow in the ED [12]. The SBU included evaluation of triage systems for use in somatic adult patients. The authors claimed that there was insufficient scientific documentation to decide whether triage scales are reproducible, and also whether the studied triage scales (METTS, ADAPT, MTS) differs concerning safety, reliability and reproducibility [12]. The review indicated that patients with less acute need for care as assessed by triage seemed to be less likely to die within short time compared to patients whose need for acute care were higher as assessed with the same triage system. The effect of triage systems used in the pre-hospital setting was not evaluated, nor was the effects of implementing the same triage system in two or more settings of the EMS [12]. Another systematic review [13] asked whether triage systems across a broad spectrum of health services affect patient flow. The authors found conflicting evidence on improvement in overall patient flow with the use of triage systems that only prioritize patients, without providing any treatment. Pre-hospital emergency triage was not a subject of the review.

In the Norwegian ambulance services, some of the district ambulance services have implemented validated triage methods or are about to do so [14]. Research that evaluates the effect of pre-hospital triage systems might influence the choice of which triage system to implement. This systematic review aims to identify and critically review literature that evaluates the effect of validated triage systems for use in the pre-hospital setting on health outcomes, patient safety, patient satisfaction, satisfaction with the use of the triage system(s), resource use, goal achievement, and quality of the information exchange between the different settings of the EMS (for example the quality of documentation). The specific research questions are: 1) are triage systems, used in the pre-hospital setting, effective; 2) is one triage system more effective than others; and 3) is it effective to use the same triage system in two or more settings of the acute chain? The current systematic review is an update of our Norwegian report raising the same research questions [15].

## Methods

The systematic literature search was conducted in June, 2012 (week 22). MEDLINE, Cinahl, EMBASE, PsycINFO, Cochrane Database of Systematic Reviews, Cochrane Central Register of Controlled Trials (CENTRAL), British nursing index (BNI), DARE via CRD and HTA via CRD were systematically searched. We applied no restrictions considering publication language. Full details of the search terms used in MEDLINE are shown in Additional file 1, and the search details for all databases are shown in the Additional file in our Norwegian report [15]. The search was complemented by a search in reference lists in review articles. We also contacted experts in Norway.

Two persons independently read titles and abstracts to identify possibly relevant articles, and the same persons independently read the full text version of possibly relevant articles identified. Any disagreement was settled by discussion or by involving a third person. We evaluated the relevance of selected articles based on the inclusion and exclusion criteria listed in Table 1. The quality judgment of the potentially relevant systematic reviews identified in the search was assessed by two persons independently using a standard checklist (Additional file 2) based on information on search strategy, inclusion criteria, selection bias, assessment of intern validity, assessment of methodological quality of included studies, how results were summarized and if the conclusions were in accordance with extracted data.

**Table 1:** Inclusion and exclusion criteria

	
Study population	Patients of all age ages in the need for acute care (acutely ill or seriously injured somatic or psychiatric patients)
Intervention	Patient prioritizing by the use of a validated triage system in the pre-hospital setting; face-to-face or telephone triage-assessment
Comparison	Acutely ill or seriously injured patients who were assessed with a triage system different from that of the intervention, or who were not triaged at all in the same type of setting
Outcomes	Health outcomes (mortality, morbidity)
	Patient safety (for example undertriage)
	Patient satisfaction
	Job-satisfaction with the triage systems among health workers
	Resources use (for example overtriage)
	To what degree triage was completed (goal achievement)
	The quality of the information exchange between the different settings of the EMS (for example the quality of documentation)
Study design	Systematic review of high quality (see checklist, Additional file 2)
	Randomized controlled trial (RCT)
	Non-randomized controlled study (non-RCT)
	Controlled before-and-after study (CBA)
	Interrupted time series analysis (ITS)
Exclusion	Studies were excluded if triage assessment was done in the hospital setting only without including triage assessment in any of the pre-hospital settings, if the patients were not acutely ill or seriously injured, or if there was no use of a comparison for the evaluation of the effects of a triage system or an ITS design

For this review of the literature, we planned to assess the methodological quality of included studies [16], to summarize their results by a descriptive synthesis or with meta-analyses by the use of Review Manager 5.1 [17] where appropriate, and to assess to which degree we could have confidence in results by the use of the GRADE methodology (Grading of Recommendations, Assessment, Development, and Evaluation) [18].

## Results

The literature search identified 13959 titles, and after removing duplicates, 11011 unique references. The distribution of the references were as follows: in MEDLINE and EMBASE 9981 hits, Cinahl 1529 hits, PsycINFO 617 hits, Cochrane Database of Systematic Reviews 8 hits, Cochrane Central Register of Controlled Trials (CENTRAL) 466 hits, British nursing index (BNI) 487 hits, DARE via CRD 757 hits and HTA via CRD 114 hits.

**Figure 1:**
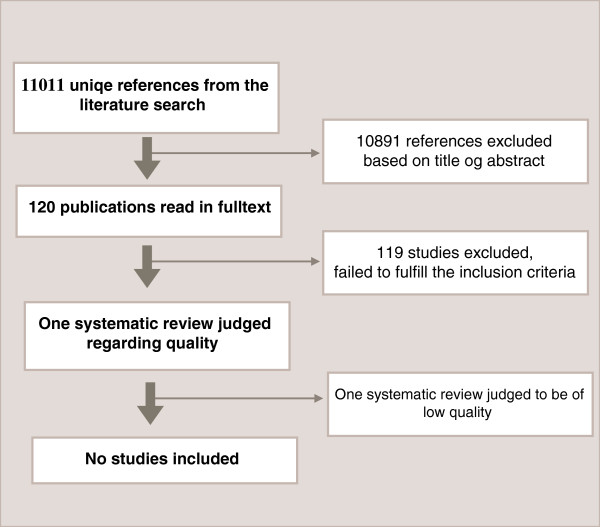
Flow chart of the literature search and the inclusion process.

We selected 120 publications for evaluation in full text (Figure 1). The final selection was based on relevance according to our inclusion criteria. Only one article, a systematic review [19], was considered relevant, but its low methodological quality (see Additional file 2) did not allow for inclusion in our systematic review. Details of why studies were excluded are reported in Additional file 3: Table S1.

None of the 120 articles studied in full text fulfilled all our inclusion criteria. Several of these articles failed to fulfill two or more of our inclusion criteria. More than half (n = 66) of the studies were excluded because they did not evaluate triage systems. In another 26 studies, the main reason for exclusion was the study design. Twenty-two studies were excluded since the study-setting did not include pre-hospital triage, and the remaining publications were excluded because they were not based on a scientific study or because they did not deal with acutely ill or seriously injured patients.

## Discussion

The main finding of this systematic review is that there is a lack of scientific documentation evaluating whether or not pre-hospital triage systems are effective, if one triage system is more effective than others, and whether or not it is effective to use the same triage system in two or more settings of an EMS regarding health outcomes, patient safety, patient satisfaction, user-friendliness, resource use, goal achievement, and regarding the quality of the information flow between the different settings of the EMS. Although we conducted a broad systematic search in several databases, we did not find any systematic reviews of high quality, controlled studies (RCTs, non-RCTs or CBAs) or ITSs that could answer any of the three research questions addressed in this systematic review.

The 120 articles that we selected for further evaluation in full text, dealt with various settings of the EMS and included study populations from the somatic field and the psychiatric field as well as patients of all age. However, all the publications failed to fulfill our inclusion criteria in some way.

To study effects of interventions, controlled studies or ITS designs are needed [20]. Many of the full text articles we evaluated, did not use any of these designs, thus an assessment of effect cannot possible be drawn. It has been questioned whether an RCT design is suitable for studies conducted in the EMS pre-hospital setting [21]. Recently, in The New England Journal of Medicine [21], examples of situations where it may be difficult or ethically unjustifiable to conduct an RCT were given. When it comes to the study of triage systems, however, we claim that it should be possible to carry out studies with control groups. A suggestion could be to compare outcomes of the intervention (the use of a triage system) with the neighboring municipality / neighboring region that have not introduced a triage system or have chosen a different triage system, or by the use of an ITS.

One of the studies identified in our search is a good example that controlled trials are feasible in this field: Ortolani et al. studied the usefulness of pre-hospital triage with regard to treatment delay and mortality in patients with ST-elevation myocardial infarction (STEMI) complicated by cardiogenic shock referred for primary percutaneous coronary intervention [22]. They conducted a controlled study by the use of differently equipped ambulances, one type ambulances equipped for triage (intervention) and one type without triage facilities (control). The triage procedure was described as to diagnose myocardial infarction by the use of technical equipment available in the ambulance, such as electrocardiogram (ECG) and to communicate with a cardiologist via a mobile communication network. The reason why we excluded Ortolani’s study from this systematic review was that the authors did not report use of a validated triage system.

Many publications were excluded since they did not study pre-hospital triage or triage in the primary care setting, but triage in-hospital. The SBU has summarized and evaluated the evidence on the effect of triage systems in EDs [12]. In their review, based on the original report [23], focusing on triage related interventions to improve patient flow in EDs, the authors call for more attention to processes outside the ED including processes before the ED, i.e. in primary care and in pre-hospital settings. The authors also underlined a need for studies evaluating processes after the ED stay, like provision of hospital beds. The SBU report emphasized the interlaced relationship between processes *before*, *in* and *after* ED stay.

The use of triage systems are about to be introduced in all parts of the EMS in the Scandinavian countries. The aims are to improve the initial assessment of patients in need of acute care, to manage resources and to increase the quality of professional prioritizing of patients. The idea of good patient flow is also central. The choice of which triage system to implement, should be evidence based. The fact that there are no published studies on the effect of triage systems used to determine the level of urgency in the pre-hospital settings is an important finding itself. It does not mean that triage does not work or that triage is ineffective. However, we cannot say how effective triage systems are and if one system is superior to another. Thus, we need well planned controlled studies or ITSs on the effects of out of hospital triage systems.

We underline that although we did not find relevant studies evaluating the effect of pre-hospital triage systems or the effect of using the same triage systems in two or more settings of the EMS, it is of course possible that there are studies that our search did not capture. We conducted a broad literature search, however we cannot be certain that we used the best suitable search-terms and MeSH (Medical Subject Headings)-terms (Additional file 1).

Scientific approaches with different study designs have the potential to inform the choice of a triage system - in different ways. However, research on effects of an intervention is best studied by undertaking RCTs because they are more likely to provide unbiased information than other study designs [20]. We extended our systematic review to include publications with the following additional designs: non-RCTs, CBAs and ITSs and still, we did not find any studies to include.

The current systematic review is an update of our Norwegian report raising the same research questions [15]. Although the results are already available in Norwegian, we claim that it is important to publish the results in an international journal because of the following reasons: 1)It is important to update the review to include potential new studies, 2) It is important to inform the field of trauma and emergency medicine that studies on the effect of pre-hospital triage systems are needed, also to those who do not read Norwegian, 3) To highlight the importance of well-planned studies minimizing or avoiding the risk of bias, 4) To inform decision makers that current knowledge on pre-hospital triage-systems lack scientific evidence on effect, 5) To avoid duplication of effort.

## Conclusion

From this systematic review, we conclude that there is a lack of scientific evidence about the effects of validated pre-hospital triage systems and about the effects of using the same triage system in two or more settings of the EMS. The fact that there is no robust evidence on the effect of pre-hospital triage systems does not mean that such systems are ineffective. It means that we do not know whether the systems are effective, nor can we suggest the size of a potential effect. When introducing a new assessment tool in the EMS, it is timely to conduct a study. In the case of a pre-hospital triage system, we emphasize the importance of well-planned studies aimed to assess effect, such as RCTs, cluster RCTs, controlled before-and-after studies or interrupted time series analysis with three observations before and after the triage-intervention.

## Competing interests

All three authors declare that we have no competing interests.

## Authors’ contributions

All three authors (IBL, HHH and GEV) planned and designed this systematic review. IBL and HHH independently read titles and abstracts to identify possibly relevant articles, and read the full text version of possibly relevant articles. IBL and HHH assessed the relevance of selected articles. Any disagreement was settled by discussion or by involving GEV. All three authors participated with writing the manuscript. All authors read and approved the final manuscript.

## Supplementary Material

Additional file 1 Search strategy. Click here for file

Additional file 2 Checklist for quality assessment of systematic reviews. Click here for file

Additional file 3: Table S1 List of excluded studies. Click here for file
