# Supplementary material for: Triage systems for pre-hospital emergency medical services - a systematic review
Source: Scand J Trauma Resusc Emerg Med. 2013 Apr 15;21:28. doi: 10.1186/1757-7241-21-28 (PMC3641954; doi:10.1186/1757-7241-21-28)
Supplement: Additional file 1 — Search strategy. [file 1757-7241-21-28-S1.doc]

Additional file 1; Search strategy

*EMBASE 1980 to 2011 Week 22, Ovid MEDLINE(R) 1948 to June Week 1 2011 (total hits: 7581)*

| 1 | Triage/ |
| --- | --- |
| 2 | Hotlines/ |
| 3 | apache/ |
| 4 | or/1-3 |
| 5 | Emergency Medical Services/ |
| 6 | exp Emergency Service, Hospital/ |
| 7 | Emergency Services, Psychiatric/ |
| 8 | Emergency Medicine/ |
| 9 | Disasters/ |
| 10 | Emergencies/ |
| 11 | mass casualty incidents/ |
| 12 | Rescue Work/ |
| 13 | or/5-12 |
| 14 | 4 and 13 |
| 15 | Triage/mt [Methods] |
| 16 | Emergency Medical Service Communication Systems/ |
| 17 | ((triag* or referral?) adj2 (system or systems or tool? or method?)).ti,ab. |
| 18 | ((triag* or referral? or overtriag* or undertriag* or priorit* of patient* or priorit* patient* or patient sorting or sorting of patient* or classify* patient* or telephon* counsel* or phon* counsel* or telephon* consult* or phon* consult* or helpline? or hotline?) and (emergen* or acute* or accident* or casuality or casualities or catastroph* or disaster* or war)).ti. |
| 19 | ((triag* or referral? or overtriag* or undertriag* or priorit* of patient* or priorit* patient* or patient sorting or sorting of patient* or classif* patient* or telephon* counsel* or phon* counsel* or telephon* consult* or phon* consult* or helpline? or hotline?) adj6 (emergen* or acute* or accident* or casuality or casualities or catastroph* or disaster* or war)).ab. |
| 20 | or/15-19 |
| 21 | 14 or 20 |
| 22 | randomized controlled trial.pt. |
| 23 | controlled clinical trial.pt. |
| 24 | (randomised or randomized).ti,ab. |
| 25 | clinical trials as topic/ |
| 26 | randomly.ti,ab. |
| 27 | trial.ti,ab. |
| 28 | intervention*.ti,ab. |
| 29 | evaluat*.ti,ab. |
| 30 | control*.ti,ab. |
| 31 | effect?.ti,ab. |
| 32 | impact.ti,ab. |
| 33 | (time series or time points).ti,ab. |
| 34 | ((pre test or pretest) and (post test or posttest)).ti,ab. |
| 35 | (quasi experiment* or quasiexperiment*).ti,ab. |
| 36 | ((multicenter or multicentre or multi center or multi centre) adj study).ti,ab. |
| 37 | or/22-36 |
| 38 | “comment on”.cm. |
| 39 | (editorial or comment or news).pt. |
| 40 | exp Animals/ not Humans.sh. |
| 41 | or/38-40 |
| 42 | 37 not 41 |
| 43 | 21 and 42 |
| 44 | limit 21 to “reviews (best balance of sensitivity and specificity)” |
| 45 | 43 or 44 |
| 46 | 45 use mesz |
| 47 | triage.tw. |
| 48 | telephone/ |
| 49 | apache/ |
| 50 | or/47-49 |
| 51 | emergency health service/ |
| 52 | emergency medicine/ |
| 53 | disaster/ |
| 54 | emergency/ |
| 55 | mass disaster/ |
| 56 | rescue work/ |
| 57 | or/51-56 |
| 58 | 50 and 57 |
| 59 | ((triag* or referral?) adj2 (system or systems or tool? or method?)).ti,ab. |
| 60 | ((triag* or referral? or overtriag* or undertriag* or priorit* of patient* or priorit* patient* or patient sorting or sorting of patient* or classify* patient* or telephon* counsel* or phon* counsel* or telephon* consult* or phon* consult* or helpline? or hotline?) and (emergen* or acute* or accident* or casuality or casualities or catastroph* or disaster* or war)).ti. |
| 61 | ((triag* or referral? or overtriag* or undertriag* or priorit* of patient* or priorit* patient* or patient sorting or sorting of patient* or classif* patient* or telephon* counsel* or phon* counsel* or telephon* consult* or phon* consult* or helpline? or hotline?) adj6 (emergen* or acute* or accident* or casuality or casualities or catastroph* or disaster* or war)).ab. |
| 62 | or/59-61 |
| 63 | 58 or 62 |
| 64 | randomized controlled trial/ |
| 65 | controlled study/ |
| 66 | (randomised or randomized).ti,ab. |
| 67 | exp “clinical trial (topic)”/ |
| 68 | randomly.ti,ab. |
| 69 | trial.ti,ab. |
| 70 | intervention*.ti,ab. |
| 71 | evaluat*.ti,ab. |
| 72 | control*.ti,ab. |
| 73 | effect?.ti,ab. |
| 74 | impact.ti,ab. |
| 75 | (time series or time points).ti,ab. |
| 76 | ((pre test or pretest) and (post test or posttest)).ti,ab. |
| 77 | (quasi experiment* or quasiexperiment*).ti,ab. |
| 78 | ((multicenter or multicentre or multi center or multi centre) adj study).ti,ab. |
| 79 | or/64-78 |
| 80 | (editorial or comment).pt. |
| 81 | nonhuman/ |
| 82 | animal/ |
| 83 | human/ |
| 84 | 82 not (82 and 83) |
| 85 | or/80-81,84 |
| 86 | 79 not 85 |
| 87 | 63 and 86 |
| 88 | limit 63 to “reviews (best balance of sensitivity and specificity)” |
| 89 | 87 or 88 |
| 90 | 89 use emez |
| 91 | 46 or 90 |
| 92 | limit 91 to yr = “2005 -Current” |
| 93 | 91 not 92 |
| 94 | remove duplicates from 92 |
| 95 | remove duplicates from 93 |
| 96 | 94 or 95 |
