# Supplementary material for: Triage systems for pre-hospital emergency medical services - a systematic review
Source: Scand J Trauma Resusc Emerg Med. 2013 Apr 15;21:28. doi: 10.1186/1757-7241-21-28 (PMC3641954; doi:10.1186/1757-7241-21-28)
Supplement: Additional file 2 — Checklist for quality assessment of systematic reviews. [file 1757-7241-21-28-S2.docx]

**Additional file 2;** Checklist for quality assessment of systematic reviews

## Checklist for systematic reviews; completed checklist for Kilner et al (19)

|  | Yes | Unclear | No |
| --- | --- | --- | --- |
| 1. Do the authors clearly describe the methods used to find the primary studies? | x |  |  |
| *Comment:* | |  |  |
| 2. Was the literature search reasonably comprehensive? |  | x |  |
| *Comment:* The search strategy appears insufficient | |  |  |
| 3. Do the authors describe the criteria used when choosing studies for inclusion (study design, participants, intervention, outcomes)? |  | x |  |
| *Comment:* The authors included studies reporting on the psychometric quality of triage-tools aimed for the use in pre-hospital trauma patients. It is unclear which study designs that were relevant for the review. | | | |
| 4. Did the authors use appropriate criteria to assess the risk for bias in the inclusion process (the use of explicit selection criteria, independent assessments by two or more persons)? | x |  |  |
| *Comment:* | |  |  |
| 5. Is a set of criteria to determine intern validity described? |  | x |  |
| *Comment:* The International Liaison Committee on Resuscitation (ILCOR) was used to determine the “level of evidence: good/fair/poor”. However none of the included studies had used a control-group. | | | |
| 6. Is the validity of included studies evaluated with relevant criteria? |  | x |  |
| *Comment:* There is no information on this. | |  |  |
| 7. Are the methods used for analysing the findings of the included studies, clearly described? |  | x |  |
| *Comment:* | |  |  |
| 8. Were the data and results of the included studies reliably reported? | x |  |  |
| *Comment:* | |  |  |
| 9. Were the authors conclusions supported by data and/or the analyse reported in the review? | x |  |  |
| *Comment:* | |  |  |
| 10. Overall - how would you rate the scientific quality of this review? | High | Medium | Low |
| *Comment:* This review does not evaluate effects of triage-tools. However, it was considered for inclusion because the review assessed the psychometric quality of triage-tools. Our conclusion is that the methodological quality of the review is low. | | | |
