# Supplementary material for: Triage systems for pre-hospital emergency medical services - a systematic review
Source: Scand J Trauma Resusc Emerg Med. 2013 Apr 15;21:28. doi: 10.1186/1757-7241-21-28 (PMC3641954; doi:10.1186/1757-7241-21-28)
Supplement: Additional file 3: Table S1 — List of excluded studies. [file 1757-7241-21-28-S3.docx]

**Table 2** List of excluded studies

| **Study reference** | **Reason for exclusion** | |
| --- | --- | --- |
| Allo (1) 2009 | | Hospital study only |
| Anonymous (2) 1999 | | Not a study, but description of a study by Salk et al which has been considered separately (see below) |
| Arendts (3) 2006 | | Hospital study only |
| Asaro (4) 2008 | | Hospital study only |
| Aylwin (5) 2006 | | No triage system comparison/evaluation |
| Bengelsdorf (6) 1984 | | No triage system comparison/evaluation |
| Benner (7) 2006 | | No triage system comparison/evaluation |
| Boeke (8) 2010 | | No triage system evaluation |
| Bonin (9) 2010 | | No triage system evaluation |
| Bouillon (10) 1992 | | No triage system comparison. Study design not appropriate for inclusion in this review |
| Brillman (11) 1996 | | Hospital study only |
| Brown (12) 2006 | | No triage system comparison/evaluation. Not relevant design |
| Budd (13) 2007 |  | No triage system evaluation |
| Bunn (14) 2009 |  | Systematic review on telephone consultation, not comparison of triage systems |
| Champion (15) 1988 |  | No triage system comparison/evaluation |
| Clemmer (16) 1985 |  | Not relevant study design |
| Considine (17) 2000 |  | Hospital study only |
| Considine (18) 2007 |  | No triage system comparison, not relevant study design |
| Cooke (19) 1999 |  | No triage system comparison/evaluation; not relevant study design |
| Crouch (20) 1998 |  | No triage system comparison, not relevant study design |
| Crouch (21) 1998 |  | No triage system comparison/evaluation |
| Custer (22) 2003 |  | No triage system comparison/evaluation |
| Dale (23) 2004 |  | Not acute ill patients |
| De Luca (24) 2009 |  | No triage system evaluation |
| Dieker (25) 2010 |  | No triage system comparison/evaluation |
| Dowd (26) 2000 |  | Hospital study only |
| Emerman (27) 1991 |  | Not relevant study design |
| Engum (28) 2000 |  | Hospital study only |
| Feddersen (29) 1983 |  | No triage system evaluation |
| Feldman (30) 2006 |  | Study design |
| Feliciano (31) 1998 |  | Not relevant study design. No triage system evaluation |
| Fernandes (32) 2005 |  | Not relevant study design |
| Flynn (33) 2006 |  | No triage system comparison/evaluation |
| Franco (34) 1997 |  | No triage system evaluation |
| Fries (35) 1994 |  | No triage system evaluation |
| Furnival (36) 1999 |  | No triage system evaluation. Not relevant study design |
| Gabbe (37) 2005 |  | No triage system comparison/evaluation. Not relevant design |
| Garner (38) 2001 |  | Article included in Kilner et al. To measure the accuracy of triage algorithms. Study design: Does not include a control group for evaluation |
| Garnett (39) 2010 |  | Not a study |
| Gebhart (40) 2007 |  | Article included in Kilner et al. Study design: Does not include a control group for evaluation |
| Gladstone (41) 2009 |  | Prehospital stroke protocol, not comparison of triage system |
| Gravel (42) 2008 |  | Hospital study only |
| Gray (43) 1997 |  | No triage system comparison/evaluation; hospital study |
| Griffin (44) 1985 |  | Not a study |
| Haas (45) 2010 |  | No triage system comparison/evaluation |
| Hedges (46) 1987 |  | Not relevant study design |
| Henry (47) 1996 |  | No triage system comparison/evaluation; study design |
| Heward (48) 2004 |  | No triage system comparison/evaluation |
| Hildebrandt (49) 2006 |  | No triage system comparison/evaluation |
| Hjalte (50) 2007 |  | No triage system evaluation |
| Holcomb (51) 2005 |  | No triage system comparison/evaluation |
| Hong (52) 2008 |  | Article included in Kilner et al. Study design: Does not include a control group for evaluation |
| Husum (53) 2003 |  | Article included in Kilner et al. Study design: Does not include a control group for evaluation |
| Jacobs (54) 1984 |  | No triage system comparison/evaluation, but studying effects of Advanced Life Support prehospital trauma care compared to Basic Life Support treatment |
| Jeena (55) 2008 |  | Hospital study only |
| Kahn (56) 2009 |  | Article included in Kilner et al. Study design: Does not include a control group for evaluation |
| Kane (57) 1985 |  | Excluded due to study design |
| Karsteadt (58) 1994 |  | No triage system evaluation |
| Kerr (59) 2010 |  | No triage system comparison/evaluation |
| Kevin (60) 2002 |  | A non-systematic review |
| Kilner (61) 2011 |  | Systematic review. The review was assessed as low quality. All the original articles included in Kilner's review were checked concerning relevance, and none of them were relevant for the current study |
| Kirves (62) 2010 |  | No triage system evaluation |
| Koehler (63) 1987 |  | Hospital study only |
| Kwon (64) 2007 |  | No triage system comparison/evaluation |
| Lammers (65) 1995 |  | No triage system evaluation |
| Lavoie (66) 2010 |  | Not relevant study design |
| Leach (67) 2008 |  | Article included in Kilner et al. To determine the agreement between procedural and nonprocedural outcome measures in a population of serious injured. Study design: Does not include a control group for evaluation |
| Lerner (68) 2008 |  | A non-systematic review; proposed national guideline |
| Lerner (69) 2006 |  | A non-systematic review on field triage criteria |
| Lossius (70) 2000 |  | Not relevant study design |
| Ma (71) 1999 |  | No triage system comparison/evaluation, not relevant design |
| Mackenzie (72) 2008 |  | No triage system evaluation |
| Marks (73) 2002 |  | Not comparison of triage system; not relevant design |
| Matthys (74) 2009 |  | No triage system evaluation |
| Maynard (75) 1995 |  | A paper that refers to the Myocaridal Infarction Triage and Intervention prehospital trial and its associated registry. No triage system comparison |
| Morgan (76) 2000 |  | Not acute ill patients; not relevant design |
| Morris (77) 1986 |  | The design is not relevant |
| Morrison (78) 2000 |  | No triage system comparison/evaluation; not relevant design |
| Mulholland (79) 2005 |  | Not comparison of triage system; not relevant design |
| Newgard (80) 2010 |  | Article included in Kilner et al. To assess whether more restrictive physiologic criteria would improve the specificity of ACSCOT "step 1" triage step without missing high-risk patients. Study design: Does not include a control group for evaluation |
| Norcross (81) 1995 |  | No triage system comparison, and study design not appropriate for inclusion in this review |
| Ocak (82) 2009 |  | No control group for the evaluation of the ACSCOT |
| Ochsner (83) 1995 |  | Hospital study only |
| Ong (84) 2008 |  | No triage system evaluation. Not acute ill patients |
| Ortolani (85) 2007 |  | No description of a triage system. The study compared patients who were transported by ambulances equipped or not equipped for triage (here: diagnostics of cardiac infarction). |
| Paul (86) 2009 |  | No triage system evaluation |
| Pedersen (87) 2009 |  | No triage system comparison/evaluation |
| Plaisier (88) 1998 |  | Study design not appropriate for inclusion in this review |
| Pointer (89) 2001 |  | No triage system evaluation |
| Purtill (90) 2008 |  | The study design not appropriate for inclusion in this review |
| Qazi (91) 1998 |  | Hospital study only |
| Quain (92) 2008 |  | Pre-hospital Acute Stroke Triage; used historical controls. |
| Rainer (93) 1997 |  | No triage system evaluation |
| Roorda (94) 1996 |  | The study design not appropriate for inclusion in this review |
| Rowlands (95) 2003 |  | No triage system evaluation |
| Sacco (96) 2005 |  | Article included in Kilner et al. Mathematical formulation of resource-constrained triage |
| Sacco (97) 2007 |  | Article included in Kilner et al. Mathematical formulation of resource-constrained triage |
| Salk (98) 1988 |  | No triage system evaluation |
| Sarikaya (99) 2004 |  | Hospital study only, not triage system comparison |
| Sartorius (100) 2010 |  | No triage system comparison. Development of the MGAP score |
| Scheetz (101) 2011 |  | No triage system comparison/evaluation |
| Simmons (102) 1995 |  | No triage system evaluation, not relevant study design |
| Sivagangabalan (103) 2009 |  | No triage system comparison/evaluation |
| Sola (104) 1994 |  | Hospital study only |
| Sporer (105) 2010 |  | No triage system comparison/evaluation |
| Stacey (106) 2003 |  | No triage system comparison/evaluation |
| Sukumaran (107) 2005 |  | No triage system comparison/evaluation |
| Taylor (108) 1996 |  | Hospital study. No triage system evaluation |
| Uleberg (109) 2007 |  | No triage system comparison/evaluation. Hospital study |
| van Ierland (110) 2011 |  | Hospital study only |
| van 't Hof (111) 2006 |  | No triage system comparison/evaluation; hospital study only |
| van Veen (112) 2009 |  | Hospital triage only |
| Verma (113) 2010 |  | No triage system comparison/evaluation |
| Wallis (114) 2006 |  | Article included in Kilner et al. To validate the Paediatric triage tape as a triage tool. Study design not appropriate for inclusion in this review |
| Wallis (115) 2006 |  | Article included in Kilner et al. To determine the sensitivity and specificity of paediatric major incidents triage scores. Study design not appropriate for inclusion in this review |
| Wallis (116) 2006 |  | No triage system comparison/evaluation |
| Wong (117) 2000 |  | Not triage system comparison, hospital level only |
| Yousif (118) 2005 |  | Hospital study only |
| Youssef (119) 2000 |  | No triage system comparison/evaluation |

Reference List

1. Allo JC, Vigneau JF, Jiang J, Ranerison R, Caroline E, Dabreteau A, et al. Appropriateness of diagnosis and orientation of 996 consecutive patients admitted in an emergency department with flow-based organization. Eur J Emerg Med 2009;16(1):23-8.

2. Telephone, in-person triage of same patients often don't agree, UCLA study concludes. Data strategies and benchmarks : the monthly advisory for health care executives 1999;3(6):94-5.

3. Arendts G, Elgafi S. Announcing the emergent patient in the emergency department: A randomised trial. EMERG MED J 2006;23(5):388-90.

4. Asaro PV, Lewis LM. Effects of a triage process conversion on the triage of high-risk presentations. Acad Emerg Med 2008;15(10):916-22.

5. Aylwin CJ, Konig TC, Brennan NW, Shirley PJ, Davies G, Walsh MS, et al. Reduction in critical mortality in urban mass casualty incidents: analysis of triage, surge, and resource use after the London bombings on July 7, 2005. Lancet 2006;368(9554):2219-25.

6. Bengelsdorf H, Levy LE, Emerson RL, Barile FA. A crisis triage rating scale. Brief dispositional assessment of patients at risk for hospitalization. J Nerv Ment Dis 1984;172(7):424-30.

7. Benner JP, Brauning G, Green M, Caldwell W, Borloz MP, Brady WJ. Disagreement between transport team and ED staff regarding the prehospital assessment of air medically evacuated scene patients. Air Med J 2006;25(4):165-9.

8. Boeke AJP, Van Randwijck-Jacobze ME, De Lange-Klerk EMS, Grol SM, Kramer MHH, Van Der Horst HE. Effectiveness of GPs in accident and emergency departments. Br J Gen Pract 2010;60(579):e378-e387.

9. Bonin JP, Dominique T, Lesage A, Fortier M, Roch L, Vadeboncoeur A, et al. Mental health. Difficulties in evaluation in triage. Perspective infirmiere : revue officielle de l'Ordre des infirmieres et infirmiers du Quebec 2010;7(1):42-8.

10. Bouillon B, Kramer M, Lechleuthner A, Tiling T. Multiple trauma: preclinical needs, transportation, time sequences. Unfallchirurgie 1992;18(2):85-90.

11. Brillman JC, Doezema D, Tandberg D, Sklar DP, Davis KD, Simms S, et al. Triage: Limitations in predicting need for emergent care and hospital admission. Ann Emerg Med 1996;27(4):493-500.

12. Brown E, Bleetman A. Ambulance alerting to hospital: the need for clearer guidance. EMERG MED J 2006;23(10):811-4.

13. Budd HR, Almond LM, Porter K. A survey of trauma alert criteria and handover practice in England and Wales. EMERG MED J 2007;24(4):302-4.

14. Bunn F, Byrne G, Kendall S. Telephone consultation and triage: effects on health care use and patient satisfaction. Cochrane database of systematic reviews (Online) 2009;(1):CD004180.

15. Champion HR, Sacco WJ, Gainer PS, Patow SM. The effect of medical direction on trauma triage. Journal of Trauma-Injury Infection and Critical Care 1988;28(2):235-9.

16. Clemmer TP, Orme J, Thomas F, Brooks KA. Prospective evaluation of the CRAMS scale for triaging major trauma. J Trauma 1985;25(3):188-91.

17. Considine J, Ung L, Thomas S. Triage nurses' decisions using the National Triage Scale for Australian emergency departments. Accid Emerg Nurs 2000;8(4):201-9.

18. Considine J, Botti M, Thomas S. Do Knowledge and Experience Have Specific Roles in Triage Decision-making? Acad Emerg Med 2007;14(8):722-6.

19. Cooke MW, Morrell R, Wilson S, Bridge P, Edwards S, Allan TF, et al. Does criteria based dispatch of 999 calls adequately detect the critically ill and injured? Pre-hospital Immediate Care 1999;3(4):191-5.

20. Crouch R, Dale J. Telephone triage--identifying the demand (Part 1). Nurs Stand 1998;12(34):33-8.

21. Crouch R, Dale J. Telephone triage--how good are the decisions? (Part 2). Nurs Stand 1998;12(35):33-9.

22. Custer M, O'Rourke KM, Roddy M, King R, Sprinkle L, Horne E. The impact of a nursing triage line on the use of emergency department services in a military hospital. Mil Med 2003;168(12):981-5.

23. Dale J, Williams S, Foster T, Higgins J, Snooks H, Crouch R, et al. Safety of telephone consultation for "non-serious" emergency ambulance service patients. Quality and Safety in Health Care 2004;13(5):363-73.

24. De LA, Toni D, Lauria L, Sacchetti ML, Giorgi RP, Ferri M, et al. An emergency clinical pathway for stroke patients--results of a cluster randomised trial (isrctn41456865). BMC health services research 2009;9:14.

25. Dieker H-J, Liem SSB, El AH, Van GP, Aengevaeren WRM, Brouwer MA, et al. Pre-hospital triage for primary angioplasty: Direct referral to the intervention center versus interhospital transport. JACC: Cardiovascular Interventions 2010;3(7):705-11.

26. Dowd MD, McAneney C, Lacher M, Ruddy RM. Maximizing the sensitivity and specificity of pediatric trauma team activation criteria. Acad Emerg Med 2000;7(10):1119-25.

27. Emerman CL, Shade B, Kubincanek J. A comparison of EMT judgment and prehospital trauma triage instruments. J Trauma 1991;31(10):1369-75.

28. Engum SA, Mitchell MK, Scherer LR, Gomez G, Jacobson L, Solotkin K, et al. Prehospital triage in the injured pediatric patient. J Pediatr Surg 2000;35(1):82-7.

29. Feddersen A. Trauma care. Evaluating effectiveness with the injury severity score. Journal of Emergency Medical Services 1983;8(9):42-4.

30. Feldman MJ, Verbeek PR, Lyons DG, Chad SJ, Craig AM, Schwartz B. Comparison of the medical priority dispatch system to an out-of-hospital patient acuity score. Acad Emerg Med 2006;13(9):954-60.

31. Feliciano DV, Anderson J, Rozycki GS, Ingram WL, Ansley JP, Namias N, et al. Management of casualties from the bombing at the Centennial Olympics. Am J Surg 1998;176(6):538-43.

32. Fernandes CM, Tanabe P, Gilboy N, Johnson LA, McNair RS, Rosenau AM, et al. Five-level triage: a report from the ACEP/ENA Five-level Triage Task Force. J Emerg Nurs 2005;31(1):39-50.

33. Flynn J, Archer F, Morgans A. Sensitivity and specificity of the medical priority dispatch system in detecting cardiac arrest emergency calls in Melbourne. Prehospital & Disaster Medicine 2006;21(2):72-6.

34. Franco SM, Mitchell CK, Buzon RM. Primary care physician access and gatekeeping: a key to reducing emergency department use. Clin Pediatr (Phila) 1997;36(2):63-8.

35. Fries GR, McCalla G, Levitt MA, Cordova R. A prospective comparison of paramedic judgment and the Trauma Triage Rule in the prehospital setting. Ann Emerg Med 1994;24(5):885-9.

36. Furnival RA, Schunk JE. ABCs of scoring systems for pediatric trauma. Pediatr Emerg Care 1999;15(3):215-23.

37. Gabbe BJ, Cameron PA, Wolfe R, Simpson P, Smith KL, McNeil JJ. Prehospital prediction of intensive care unit stay and mortality in blunt trauma patients. Journal of Trauma - Injury, Infection and Critical Care 2005;59(2):458-65.

38. Garner A, Lee A, Harrison K, Schultz CH. Comparative analysis of multiple-casualty incident triage algorithms. Ann Emerg Med 2001;38(5):541-8.

39. Garnett AR, Marsden DL, Parsons MW, Quain DA, Spratt NJ, Loudfoot AR, et al. The rural Prehospital Acute Stroke Triage (PAST) trial protocol: A controlled trial for rapid facilitated transport of rural acute stroke patients to a regional stroke centre. International Journal of Stroke 2010;5(6):506-13.

40. Gebhart ME, Pence R. START Triage: Does It Work? Disaster Management and Response 2007;5(3):68-73.

41. Gladstone DJ, Rodan LH, Sahlas DJ, Lee L, Murray BJ, Ween JE, et al. A citywide prehospital protocol increases access to stroke thrombolysis in toronto. Stroke (00392499) 2009;40(12):3841-4.

42. Gravel J, Gouin S, Manzano S, Arsenault M, Amre D. Interrater agreement between nurses for the pediatric canadian triage and acuity scale in a tertiary care center. Acad Emerg Med 2008;15(12):1262-7.

43. Gray A, Goyder EC, Goodacre SW, Johnson GS. Trauma triage: A comparison of CRAMS and TRTS in a UK population. Injury 1997;28(2):97-101.

44. Griffin GD, Hays SB. Rapid and phased evaluation of the acutely ill or traumatized patient. Mil Med 1985;150(10):534-41.

45. Haas B, Gomez D, Zagorski B, Stukel TA, Rubenfeld GD, Nathens AB. Survival of the fittest: The hidden cost of undertriage of major trauma. J Am Coll Surg 2010;211(6):804-11.

46. Hedges JR, Feero S, Moore B, Haver DW, Shultz B. Comparison of prehospital trauma triage instruments in a semirural population. The Journal of emergency medicine 1987;5(3):197-208.

47. Henry MC, Alicandro JM, Hollander JE, Moldashel JG, Cassara G, Thode J. Evaluation of American College of Surgeons trauma triage criteria in a suburban and rural setting. Am J Emerg Med 1996;14(2):124-9.

48. Heward A, Damiani M, Hartley-Sharpe C. Does the use of the advanced medical priority dispatch system affect cardiac arrest detection? EMERG MED J 2004;21(1):115-8.

49. Hildebrandt DE, Westfall JM, Fernald DH, Pace WD. Harm resulting from inappropriate telephone triage in primary care. Journal of the American Board of Family Medicine 2006;19(5):437-42.

50. Hjalte L, Suserud BO, Herlitz J, Karlberg I. Why are people without medical needs transported by ambulance? A study of indications for pre-hospital care. Eur J Emerg Med 2007;14(3):151-6.

51. Holcomb JB, Niles SE, Miller CC, Hinds D, Duke JH, Moore FA. Prehospital physiologic data and lifesaving interventions in trauma patients. Mil Med 2005;170(1):7-13.

52. Hong R, Sierzenski PR, Bollinger M, Durie CC, O'Connor RE. Does the simple triage and rapid treatment method appropriately triage patients based on trauma injury severity score? American journal of disaster medicine 2008;3(5):265-71.

53. Husum H, Gilbert M, Wisborg T, Van Heng Y, Murad M. Respiratory rate as a prehospital triage tool in rural trauma. J Trauma 2003;55(3):466-70.

54. Jacobs LM, Sinclair A, Beiser A, D'Agostino RB. Prehospital advanced life support: benefits in trauma. Journal of Trauma-Injury Infection and Critical Care 1984;24(1):8-13.

55. Jeena PM, Adhikari M, Carlin JB, Qazi S, Weber MW, Hamer DH. Clinical profile and predictors of severe illness in young South African infants (<60 days). S Afr Med J 2008;98(11):883-8.

56. Kahn CA, Schultz CH, Miller KT, Anderson CL. Does START Triage Work? An Outcomes Assessment After a Disaster. Ann Emerg Med 2009;54(3):424-30.

57. Kane G, Engelhardt R, Celentano J. Empirical development and evaluation of prehospital trauma triage instruments. J Trauma 1985;25(6):482-9.

58. Karsteadt LL, Larsen CL, Farmer PD. Analysis of a rural trauma program using the TRISS methodology: a three-year retrospective study. Journal of Trauma-Injury Infection and Critical Care 1994;36(3):395-400.

59. Kerr E, Arulraj N, Scott M, McDowall M, van DM, Keir S, et al. A telephone hotline for transient ischaemic attack and stroke: prospective audit of a model to improve rapid access to specialist stroke care. BMJ (Clinical research ed ) 2010;341:c3265.

60. Kevin J. An examination of telephone triage in a mental health context. Issues Ment Health Nurs 2002;23(8):757-69.

61. Kilner TM, Brace SJ, Cooke MW, Stallard N, Bleetman A, Perkins GD. In 'big bang' major incidents do triage tools accurately predict clinical priority?: A systematic review of the literature. Injury 2011;42(5):460-8.

62. Kirves H, Handolin L, Niemela M, Pitkaniemi J, Randell T. Paramedics' and pre-hospital physicians' assessments of anatomic injury in trauma patients: a cohort study. Scandinavian Journal of Trauma, Resuscitation and Emergency Medicine 2010;18:60.

63. Koehler JJ, Malafa SA, Hillesland J, Baer LJ, Rogers RN, Navitskas NR, et al. A multicenter validation of the prehospital index. Ann Emerg Med 1987;16(4):380-5.

64. Kwon WY, Rhee JE, Gang HS, Shin SD, Cho JH, Song HG, et al. Triage method for out-of-hospital poisoned patients. J Korean Med Sci 2007;22(2):336-41.

65. Lammers RL, Roth BA, Utecht T. Comparison of ambulance dispatch protocols for nontraumatic abdominal pain. Ann Emerg Med 1995;26(5):579-89.

66. Lavoie A, Emond M, Moore L, Camden S, Liberman M. Evaluation of the prehospital index, presence of highvelocity impact and judgment of emergency medical technicians as criteria for trauma triage. Canadian Journal of Emergency Medicine 2010;12(2):111-8.

67. Leach SR, Swor RA, Jackson RE, Fringer RC, Bonfiglio AX. Do outcome measures for trauma triage agree? Prehosp Emerg Care 2008;12(4):467-9.

68. Lerner EB, Schwartz RB, Coule PL, Weinstein ES, Cone DC, Hunt RC, et al. Mass casualty triage: an evaluation of the data and development of a proposed national guideline. Disaster medicine and public health preparedness 2008;2 Suppl 1:S25-S34.

69. Lerner EB. Studies evaluating current field triage: 1966 - 2005. Prehosp Emerg Care 2006;10(3):303-6.

70. Lossius HM, Langhelle A, Pillgram-Larsen J, Lossius TA, Soreide E, Laake P, et al. Efficiency of activation of the trauma team in a Norwegian trauma referral centre. Eur J Surg 2000;166(10):760-4.

71. Ma MH, MacKenzie EJ, Alcorta R, Kelen GD. Compliance with prehospital triage protocols for major trauma patients. Journal of Trauma-Injury Infection and Critical Care 1999;46(1):168-75.

72. Mackenzie CF, Hu P, Sen A, Dutton R, Seebode S, Floccare D, et al. Automatic pre-hospital vital signs waveform and trend data capture fills quality management, triage and outcome prediction gaps. AMIA 2008;Annual Symposium proceedings / AMIA Symposium. AMIA Symposium.:318-22.

73. Marks PJ, Daniel TD, Afolabi O, Spiers G, Nguyen-Van-Tam JS. Emergency (999) calls to the ambulance service that do not result in the patient being transported to hospital: An epidemiological study. EMERG MED J 2002;19(5):449-52.

74. Matthys J, Buylaert W, Paepe PD, Thoen A, Matthijs J-P, Sutter AD. Should the general practitioner be prepared for the patient with decreased consciousness or coma? Eur J Emerg Med 2009;16(3):163-5.

75. Maynard C, Weaver WD. Streamlining the triage system for acute myocardial infarction. Cardiol Clin 1995;13(3):311-20.

76. Morgan J, Whelan L. Triage: a retrospective evaluation. Emergency nurse : the journal of the RCN Accident and Emergency Nursing Association 2000;8(5):22-9.

77. Morris J, Auerbach PS, Marshall GA. The trauma score as a triage tool in the prehospital setting. Journal of the American Medical Association 1986;256(10):1319-25.

78. Morrison A, Hull A, Shephard B. Triage in emergency psychiatry. Psychiatric Bulletin 2000;24(7):261-4.

79. Mulholland SA, Gabbe BJ, Cameron P, Victorian State Trauma Outcomes Registry and Monitoring Group (VSTORM). Is paramedic judgement useful in prehospital trauma triage? Injury 2005;36(11):1298-305.

80. Newgard CD, Rudser K, Hedges JR, Kerby JD, Stiell IG, Davis DP, et al. A critical assessment of the out-of-hospital trauma triage guidelines for physiologic abnormality. Journal of Trauma - Injury, Infection and Critical Care 2010;68(2):452-62.

81. Norcross ED, Ford DW, Cooper ME, Zone-Smith L, Byrne TK, Yarbrough III DR. Application of American College of Surgeons' field triage guidelines by pre-hospital personnel. J Am Coll Surg 1995;181(6):539-44.

82. Ocak G, Sturms LM, Hoogeveen JM, Le CS, Jukema GN. Prehospital identification of major trauma patients. Langenbeck's Archives of Surgery 2009;394(2):285-92.

83. Ochsner MG, Schmidt JA, Rozycki GS, Champion HR. The evaluation of a two-tier trauma response system at a major trauma center: Is it cost effective and safe? Journal of Trauma - Injury, Infection and Critical Care 1995;39(5):971-7.

84. Ong RS, Post J, van RH, de HJ. Call-duration and triage decisions in out of hours cooperatives with and without the use of an expert system. BMC family practice 2008;9:11.

85. Ortolani P, Marzocchi A, Marrozzini C, Palmerini T, Saia F, Baldazzi F, et al. Usefulness of Prehospital Triage in Patients With Cardiogenic Shock Complicating ST-Elevation Myocardial Infarction Treated With Primary Percutaneous Coronary Intervention. Am J Cardiol 2007;100(5):787-92.

86. Paul AO, Kay MV, Huppertz T, Mair F, Dierking Y, Hornburger P, et al. Validation of the prehospital mSTaRT triage algorithm : A pilot study for the development of a multicenter evaluation. Unfallchirurg 2009;112(1):23-32.

87. Pedersen SH, Galatius S, Hansen PR, Mogelvang R, Abildstrom SZ, Sorensen R, et al. Field Triage Reduces Treatment Delay and Improves Long-Term Clinical Outcome in Patients With Acute ST-Segment Elevation Myocardial Infarction Treated With Primary Percutaneous Coronary Intervention. J Am Coll Cardiol 2009;54(24):2296-302.

88. Plaisier BR, Meldon SW, Super DM, Jouriles NJ, Barnoski AL, Fallon WF, Jr., et al. Effectiveness of a 2-specialty, 2-tiered triage and trauma team activation protocol. Ann Emerg Med 1998;32(4):436-41.

89. Pointer JE, Levitt MA, Young JC, Promes SB, Messana BJ, Ader MEJ. Can paramedics using guidelines accurately triage patients? Ann Emerg Med 2001;38(3):268-77.

90. Purtill MA, Benedict K, Hernandez-Boussard T, Brundage SI, Kritayakirana K, Sherck JP, et al. Validation of a prehospital trauma triage tool: a 10-year perspective. The Journal of trauma 2008;65(6):1253-7.

91. Qazi K, Kempf JA, Christopher NC, Gerson LW. Paramedic judgment of the need for trauma team activation for pediatric patients. Acad Emerg Med 1998;5(10):1002-7.

92. Quain DA, Parsons MW, Loudfoot AR, Spratt NJ, Evans MK, Russell ML, et al. Improving access to acute stroke therapies: A controlled trial of organised pre-hospital and emergency care. Med J Aust 2008;189(8):429-33.

93. Rainer TH, Houlihan KP, Robertson CE, Beard D, Henry JM, Gordon MW. An evaluation of paramedic activities in prehospital trauma care. Injury 1997;28(9-10):623-7.

94. Roorda J, Van Beeck EF, Stapert JWJL, Ten WW. Evaluating performance of the Revised Trauma Score as a triage instrument in the prehospital setting. Injury 1996;27(3):163-7.

95. Rowlands A. An evaluation of pre-hospital communication between ambulances and an accident and emergency department. J Telemed Telecare 2003;9 Suppl 1:S35-S37.

96. Sacco WJ, Navin DM, Fiedler KE, Waddell II RK, Long WB, Buckman J. Precise formulation and evidence-based application of resource-constrained triage. Acad Emerg Med 2005;12(8):759-70.

97. Sacco WJ, Navin DM, Waddell II RK, Fiedler KE, Long W.B., Buckman R.F. A new resource-constrained triage method applied to victims of penetrating injury. J Trauma 2007;63:316-25.

98. Salk ED, Schriger DI, Hubbell KA, Schwartz BL. Effect of visual cues, vital signs, and protocols on triage: A prospective randomized crossover trial. Ann Emerg Med 1998;32(6):655-64.

99. Sarikaya S, Soysal S, Karcioglu O, Topacoglu H, Tasar A. Paramedics and triage: effect of one training session on triage in the emergency department. Adv Ther 2004;21(5):329-34.

100. Sartorius D, Le MY, David JS, Rancurel E, Smail N, Thicoipe M, et al. Mechanism, glasgow coma scale, age, and arterial pressure (MGAP): a new simple prehospital triage score to predict mortality in trauma patients. Crit Care Med 2010;38(3):831-7.

101. Scheetz LJ. Trends in the accuracy of older person trauma triage from 2004 to 2008. Prehosp Emerg Care 2011;15(1):83-7.

102. Simmons E, Hedges JR, Irwin L, Maassberg W, Kirkwood J. Paramedic injury severity perception can aid trauma triage. Ann Emerg Med 1995;26(4):461-8.

103. Sivagangabalan G, Ong ATL, Narayan A, Sadick N, Hansen PS, Nelson GCI, et al. Effect of Prehospital Triage on Revascularization Times, Left Ventricular Function, and Survival in Patients With ST-Elevation Myocardial Infarction. Am J Cardiol 2009;103(7):907-12.

104. Sola JE, Scherer LR, Haller J, Colombani PM, Papa PA, Paidas CN. Criteria for safe cost-effective pediatric trauma triage: Prehospital evaluation and distribution of injured children. J Pediatr Surg 1994;29(6):738-41.

105. Sporer KA, Craig AM, Johnson NJ, Yeh CC. Does emergency medical dispatch priority predict delphi process-derived levels of prehospital intervention? Prehospital Disaster Med 2010;25(4):309-17.

106. Stacey D, Noorani HZ, Fisher A, Robinson D, Joyce J, Pong RW. Telephone triage services: systematic review and a survey of Canadian call centre programs. 2003.

107. Sukumaran S, Henry JM, Beard D, Lawrenson R, Gordon MWG, O'Donnell JJ, et al. Prehospital trauma management: A national study of paramedic activities. EMERG MED J 2005;22(1):60-3.

108. Taylor J, Lawrie S, Geddes J. Factors associated with admission to hospital following emergency psychiatric assessment. Health Bull (Edinb) 1996;54(6):467-73.

109. Uleberg O, Vinjevoll OP, Eriksson U, Aadahl P, Skogvoll E. Overtriage in trauma - what are the causes? Acta Anaesthesiol Scand 2007;51(9):1178-83.

110. van IY, van VM, Huibers L, Giesen P, Moll HA. Validity of telephone and physical triage in emergency care: The Netherlands Triage System. Fam Pract 2011;28(3):334-41.

111. van 't Hof AWJ, Rasoul S, Van De Wetering H, Ernst N, Suryapranata H, Hoorntje JCA, et al. Feasibility and benefit of prehospital diagnosis, triage, and therapy by paramedics only in patients who are candidates for primary angioplasty for acute myocardial infarction. Am Heart J 2006;151(6):1255.

112. van VM, Moll HA. Reliability and validity of triage systems in paediatric emergency care. Scandinavian Journal of Trauma, Resuscitation and Emergency Medicine 2009;17:38.

113. Verma A, Gladstone DJ, Fang J, Chenkin J, Black SE, Richard VP. Effect of online medical control on prehospital code stroke triage. Canadian Journal of Emergency Medicine 2010;12(2):103-10.

114. Wallis LA, Carley S. Validation of the paediatric triage tape. EMERG MED J 2006;23(1):47-50.

115. Wallis LA, Carley S. Comparison of paediatric major incident primary triage tools. EMERG MED J 2006;23(6):475-8.

116. Wallis PA, Gottschalk SB, Wood D, Bruijns S, de VS, Balfour C, et al. The Cape Triage Score -- a triage system for South Africa. S Afr Med J 2006;Afr.(1):53-6.

117. Wong TW, Lau CC. Profile and outcomes of patients transported to an accident and emergency department by helicopter: prospective case series. Hong Kong medical journal = Xianggang yi xue za zhi / Hong Kong Academy of Medicine 2000;6(3):249-53.

118. Yousif K, Bebbington J, Foley B. Impact on patients triage distribution utilizing the Australasian Triage Scale compared with its predecessor the National Triage Scale. EMA - Emergency Medicine Australasia 2005;17(5-6):429-33.

119. Youssef GG, Underhill TJ, Tovey C. Referral to the accident and emergency department following the use of community alarms. J Accid Emerg Med 2000;17(5):348-50.
